# Genome-Wide Association Analysis for Blood Lipid Traits Measured in Three Pig Populations Reveals a Substantial Level of Genetic Heterogeneity

**DOI:** 10.1371/journal.pone.0131667

**Published:** 2015-06-29

**Authors:** Hui Yang, Xiaochang Huang, Zhijun Zeng, Wanchang Zhang, Chenlong Liu, Shaoming Fang, Lusheng Huang, Congying Chen

**Affiliations:** Key Laboratory for Animal Biotechnology of Jiangxi Province and the Ministry of Agriculture of China, Jiangxi Agricultural University, 330045, Nanchang, China; University of Lleida, SPAIN

## Abstract

Serum lipids are associated with myocardial infarction and cardiovascular disease in humans. Here we dissected the genetic architecture of blood lipid traits by applying genome-wide association studies (GWAS) in 1,256 pigs from Laiwu, Erhualian and Duroc × (Landrace × Yorkshire) populations, and a meta-analysis of GWAS in more than 2,400 pigs from five diverse populations. A total of 22 genomic loci surpassing the suggestive significance level were detected on 11 pig chromosomes (SSC) for six blood lipid traits. Meta-analysis of GWAS identified 5 novel loci associated with blood lipid traits. Comparison of GWAS loci across the tested populations revealed a substantial level of genetic heterogeneity for porcine blood lipid levels. We further evaluated the causality of nine polymorphisms nearby or within the *APOB* gene on SSC3 for serum LDL-C and TC levels. Of the 9 polymorphisms, an indel showed the most significant association with LDL-C and TC in Laiwu pigs. But the significant association was not identified in the White Duroc × Erhualian F_2_ resource population, in which the QTL for LDL-C and TC was also detected on SSC3. This indicates that population-specific signals may exist for the SSC3 QTL. Further investigations are warranted to validate this assumption.

## Introduction

Blood lipids reflect lipid metabolism of the whole body and the health status of humans. Abnormal concentrations of blood lipids are associated with familial hypercholesterolemia, cardiovascular disease and diabetes. The clinical tests of total cholesterol (TC), low-density lipoprotein cholesterol (LDL-C), high-density lipoprotein (HDL-C), triglycerides (TG) and atherosclerosis index (AI) are widely used in the cardiovascular disease risk assessment [1, 2]. Identifying causal genes regulating blood lipid levels will contribute to the prevention and treatment of atherosclerosis. So far, more than 100 quantitative trait loci (QTL) have been detected to associate with one or more serum lipid contents in humans. For example, 43 loci were found to associate with plasma lipoprotein concentration and cholesterol content by genome-wide association study (GWAS) in 17,296 women [3]. Several genes, such as *GALNT2*, *TRIB1* and *SORT1*, are reported to be the causative genes for TG, very low-density lipoprotein (VLDL) and LDL-C, respectively [4–6]. However, these genetic variants together account for ~25-30% of the genetic component of phenotypic variation, suggesting that many serum lipid-associated genetic variants remain to be found [7].

Pigs have been used as a biomedical model of human disease for decades. A pig model for diabetes shows that 12-lipoxygenase and oxidant stress play key roles in accelerating atherosclerosis due to diabetes and hyperlipemia [8]. So far, more than 80 QTL for blood lipid levels have been reported in the porcine QTL database (http://www.animalgenome.org/cgi-bin/QTLdb/SS/index). We previously identified 15 QTL for serum lipids in a White Duroc × Erhualian F_2_ resource population by a genome-wide scan with 194 microsatellite markers [9]. By applying genome-wide association study (GWAS) with Illumina porcine 60K SNP beadchips, we further detected a total of 18 genomic loci for blood lipid levels in the same F_2_ resource population and a Sutai population. We found the most significant SNP in the F_2_ intercross at SSC3: 124.77 Mb for LDL-C and TC, proximal to the *APOB* gene [10, 11].

In the current study, we performed GWAS and a meta-analysis of GWAS in a total of 2,402 animals from five diverse populations, including a White Duroc × Erhualian F_2_ resource population, a Chinese synthetic commercial line of Sutai, a commercial Duroc × (Landrace × Large White) line (DLY), two Chinese indigenous pig breeds of Erhualian and Laiwu. We identified novel genomic loci associated with blood lipid contents and evaluated the associations of the *APOB* polymorphisms with blood lipids in pigs.

## Materials and Methods

### Ethic statement

All the tested animals are raised in compliance with the care and use guidelines of experimental animals established by the Ministry of Agriculture of China. This study was approved by the ethics committee of Jiangxi Agricultural University.

### Experimental populations

The experimental animals used in this study included Laiwu (N = 316), Erhualian (N = 333) and DLY (N = 607) pigs. Besides, previously reported data from the F_2_ (N = 729) and Sutai (N = 417) pigs were used in the meta-analysis of GWAS. The F_2_ and Sutai populations were described in detail in our previous studies [9, 10]. In brief, two White Duroc sires and 17 Erhualian dams were mated to produce F_1_ animals, and then nine F_1_ boars were randomly crossed with 59 F_1_ sows to produce F_2_ individuals [9]. The Sutai pig is a Chinese synthetic commercial line that was originally derived from a cross between Duroc and Erhualian breeds and has experienced artificial selection for over 18 generations. Laiwu is a Chinese indigenous pig breed that is famous for its exceptionally high intramuscular fat content (9 ~ 12%). Erhualian is another Chinese indigenous pig breed that has been known for its high prolificacy. All animals were raised in standard indoor conditions and were fed three times a day with diet containing 16% crude protein, 3100 kJ of digestible energy and 0.78% lysine. Water was available ad libitum from nipple drinkers. F_2_ and Sutai pigs were slaughtered at age of 240 ± 3 days. Laiwu and Erhualian pigs were slaughtered at 300 ± 3 days. DLY pigs were raised in a commercial company under the same feeding condition and were slaughtered at around 180 days. All experimental pigs were fasted overnight (about 12 hours) but were free access to water before slaughter.

### Phenotype recording

All blood samples were collected from the major arteries when the pigs were exsanguinated. After coagulation at room temperature, the clots were centrifuged at 3,000 rpm at 4°C for 20 min to separate serums. All serum samples were deposited at -80°C until utilized. We used the diagnostic kits of Determiner-L LDL-C, Determiner-L HDL-C, Determiner-L TG and Determiner-L TC II (Kyowa Medex, Japan) for measuring LDL-C, HDL-C, TG and TC levels, respectively, according to the manufacturer’s instructions. Atherosclerosis index (AI) was calculated according to the formula: AI = (TC-HDL-C)/HDL-C [1, 2]. All measurements were performed in an AU5421 Automatic Biochemistry Analyzer (Backman-Kelt, USA) at the First Affiliated Hospital of Nanchang University.

### Chip SNP genotyping and quality control

All experimental pigs were genotyped using Porcine SNP60 BeadChips following the Infinium HD Assay Ultra protocol (Illumina, USA). The positions of 61,565 SNPs on the chip on the current pig genome assembly (Sscrofa 10.2) were retrieved from the NRSP-8 Community Data Repository (http://www.animalgenome.org/repository/pig/Genome_build_10.2_mappings/). Quality control (QC) procedures were performed by using Plink v1.07 [12]. SNPs with call rate < 95%, minor allele frequency (MAF) < 1%, or showing significant departure from the Hardy Weinberg equilibrium (*P* < 1×10^-5^) were removed from further analysis. The same QC criteria were applied to SNP data of all tested pigs. A final set of 47,158, 33,968 and 40,152 SNPs in Laiwu, Erhualian and DLY pigs, respectively, together with 40,790 SNPs in F_2_ pigs and 49,225 SNPs in Sutai pigs, were used for subsequent statistical analyses.

### *APOB* SNP identification and genotyping

According to the *APOB* gene sequence in the pig reference genome assembly at NCBI (http://www.ncbi.nlm.nih.gov/gene), we designed 23 primer pairs (**S1 Table**) to screen exonic polymorphisms in the *APOB* gene using Primer 3 (http://primer3.ut.ee). DNA samples of two F_0_ boars and 13 F_0_ sows from the white Duroc × Erhualian F_2_ population were used to identify polymorphisms by Sanger sequencing. Amplification was performed in a 25-μl reaction mixture containing 50 ng of genomic DNA and 2 U of Taq DNA polymerase (Takara, Japan) under the thermocycle condition of 94°C for 4 minutes, 40 × (94°C for 30 sec, annealing temperature for 30 sec and 72°C for 45 sec) and 72°C for 10 minutes on a PE 9700 thermal cycler (Applied Biosystem, USA). SeqMan in the DNAStar software package (DNAStar, USA) was used to align the obtained sequences and the reference sequence. An indel and 8 single base mutations were identified on exons in the pig *APOB* gene. According to the pig *APOB* Refseq (GenBank accession: NW_003613573.1), these polymorphisms were all named after HGVS nomenclature.

We then genotyped these *APOB* polymorphisms in the five experimental populations using primers and probes listed in **S2 Table**. Eight SNPs were separately genotyped in a 10-μl reaction mixture including 30 ng of genomic DNA, 0.2 μM of each primer, 0.15 μM of each probe and 5 mM of TaqMan Genotyping Master Mix (Applied Biosystems, USA) at 95°C for 10 minutes, 40 × (95°C for 15 sec and 60°C for 1 minute) on an ABI 7900 HT (Applied Biosystems, USA). The indel was genotyped by routine PCR and agarose gel electrophoresis (**S1 Fig**). PCR products were sequenced after being purified with the QIAquick DNA Purification Kit (Qiagen, Germany) to check the sequence identity.

### Statistical analysis

#### GWAS and Meta-analysis

Heritability of each trait was estimated by using –lmm procedure of GEMMA based on genomic relationship matrix [13]. A mixed model was used to analyze the associations of the eligible SNPs with blood lipid traits: *Y = Xb + Sα+ Zμ+ e*, where *b* is the fixed effects that included sex and batch (the batch was 22, 11 and 4 for DLY, Erhualian and Laiwu, respectively); *X* is the incidence matrix of the fixed effects; *α* is the SNP substitution effect; *S* is the incidence matrix for *α*; *μ* is the vector of random additive genetic effects that follow the distribution N (0, **G**σ^2^), where G is the kinship matrix derived from SNP markers [14, 15] and σ^2^ is the additive variance; *Z* is the identity matrix for *μ*; *e* is the residual error. The mmscore function of GenABEL was used to estimate the significance of associations between SNP markers and target traits [16]. A bonferroni correction was applied to determine the genome-wide (*P* < 0.05/SNP number) and suggestive (*P* < 1/SNP number) significance thresholds. The meta-analysis of GWAS was performed on the five populations of F_2_, Sutai, DLY, Laiwu and Erhualian pigs by employing METAL [17]. In brief, for each marker, the same reference allele was selected for all tested populations and a Z-score for evidence of association was calculated. The Z-statistics summarized the magnitude and the direction of allelic effect relative to the reference allele. An overall Z-score and p-value were then calculated from a weighted sum of the individual statistics. Weights were proportional to the square-root of the number of individuals examined in each population. The porcine genome assembly 10.2 was retrieved to characterize functionally plausible candidate genes (http://www.ensembl.org/Sus_scrofa/Location/Genome).

Population stratification affects the validity of genome-wide association study [18]. Population stratification was corrected by fitting the covariance among individuals that was inferred from high density SNP data. Moreover, genomic control (GC) was used to correct the effect of stratification (λ) that was estimated from the null test statistics (under the null hypothesis of no SNP associated with the trait) [19]. Here, we evaluated population stratification by examining the distribution of test statistics in a quantile-quantile (Q-Q) plot [18]. The Q-Q plots were constructed with R software.

#### Linkage disequilibrium and linkage analysis (LDLA) for SNPs on SSC3

Haplotypes along SSC3 were reconstructed for F_2_, Laiwu and DLY pigs using the 60K SNP data, pedigree information and a Hidden Markov model [20]. The model simultaneously phased SNP genotypes and assigned the ensuing haplotypes to a predetermined number of ancestral haplotypes. Then, the effects of these ancestral haplotypes were estimated using a mixed model framework: *Y* = *Xb* + *Zu* + *e* [21], where *Y* is the vector of phenotypes; *b* is the estimator of fixed effects including sex and batch; *X* and *Z* are the incidence matrices for *b* and *u*; *u* is the random additive genetic effect following the multinormal distribution *u* ~ N (0, **G**σ_α_^2^), in which G is the individual-individual similarity matrix which was calculated from SNP information on SSC3 and σ_α_^2^ is the polygenetic additive variance; and *e* is a vector of residual error with a distribution of N(0, Iσ_e_^2^). The haplotype-based LDLA analysis can use both within-family linkage information and across-family linkage disequilibrium information resulting from historical recombination events in ancestors of founder animals. The LDLA analysis was conducted using R scripts.

#### Single marker association analysis and *F*-drop test

Single marker association test was performed for the nine polymorphisms. The genotypes of these polymorphisms were integrated into the 60K SNP genotype data of F_2_ and Laiwu pigs, respectively. The associations of these polymorphisms with blood lipids were assessed by the GWAS analysis as described above. Sex and batch were treated as fixed effects, and population substructure was corrected by fitting the kinship matrix derived from the 60K SNP data. The bonferroni correction was used to evaluate the significance threshold of the associations. In the Laiwu population, *F*-drop test was performed by separately including the genotypes of the indel, NW_003613573.1:g.20713A>G and NW_003613573.1:g.48834A>T as a fixed effect in the single marker association analysis.

## Results

### Phenotypic values

The phenotypic values of the Sutai and F_2_ populations have been reported in our previous studies [9, 10]. The phenotypic values measured in the Laiwu, DLY and Erhualian populations are listed in **S3 Table**. AI, which is set as an index to measure the degree of atherosclerosis by the international medical community, was explored for GWAS in pigs for the first time. The correlations between the six analyzed blood lipid traits are shown in S2 Fig. We estimated the heritability of these blood lipid traits, ranging from 0.12 to 0.57 in the tested populations.

### GWAS results for blood lipid levels in the three pig populations

The GWAS results of blood lipid traits in the F_2_ and Sutai populations have been shown in our previous paper [10]. In this study, we firstly performed the GWAS analysis in the Laiwu, DLY and Erhualian populations using an additive model. The “Q-Q” plots showed that the distribution of observed *P* values deviated from expected *P* values in the extreme tail (**S3 Fig**). However, the inflation factor (λ) values were around 1.0 in the three experimental populations, indicating that population structures were properly corrected.

We identified a total of 105, 9 and 8 SNPs that achieved the suggestive significance level for the measured traits in the Laiwu (*P* < 1/47,158), DLY (*P* < 1/40,152) and Erhualian (*P* < 1/33,968) populations, respectively. We found 67 SNPs that surpassed the genome-wide significance level in Laiwu pigs (*P* < 0.05/47,158). The most significant SNP across all traits was MARC0083986 (*P* = 3.25 × 10^-11^) at 125.21Mb on SSC3 in Laiwu pigs (**Table 1**). In the DLY population, only one SNP on SSC3 surpassed the genome-wide significance level for AI (*P* < 0.05/40,152). However, in Erhualian pigs, no SNPs achieved the genome-wide significance level for all six blood lipid traits (*P* > 0.05/33,968).

**Table 1:** Genome-wide significance loci identified by GWAS for serum lipids in the three tested populations.

Trait	Population	N_SNP_ ^a^	Top SNP	Position (bp)	Allele effect^b^	*P* value	Candidate gene^c^
**LDL-C**	Laiwu	39	MARC0083986	3: 125211999	0.20 ± 0.03	3.25 × 10^-11^	*NCOA1*, *KLHL29*, *APOB*, *C2ORF43*, *TTC32*
**TC**	Laiwu	27	H3GA0010711	3: 126108723	0.21 ± 0.03	2.72 × 10^-9^	*NCOA1*, *KLHL29*, *APOB*, *C2ORF43*, *TTC32*
**HDL/LDL**	Laiwu	1	MARC0010341	5: 25185108	0.24 ± 0.04	4.75 × 10^-7^	*TAC3*, *GPR182*
**AI**	DLY	1	ALGA0021166	3: 123041389	0.20 ± 0.04	2.78 × 10^-7^	*NCOA1*, *KLHL29*, *APOB*

^a^. The number of SNPs that achieved genome-wide significance level.

^b^. Allele substitution effect, the least square mean ± SE was showed for each phenotype; the unit for LDL-C and TC is mmol/L

^c^. Candidate genes were selected from annotated genes with functional relevance to blood lipids or lipid metabolism in an interval of 5 Mb centered at the top SNP at each significant locus.

#### Low density lipoproteins

**Fig 1:**
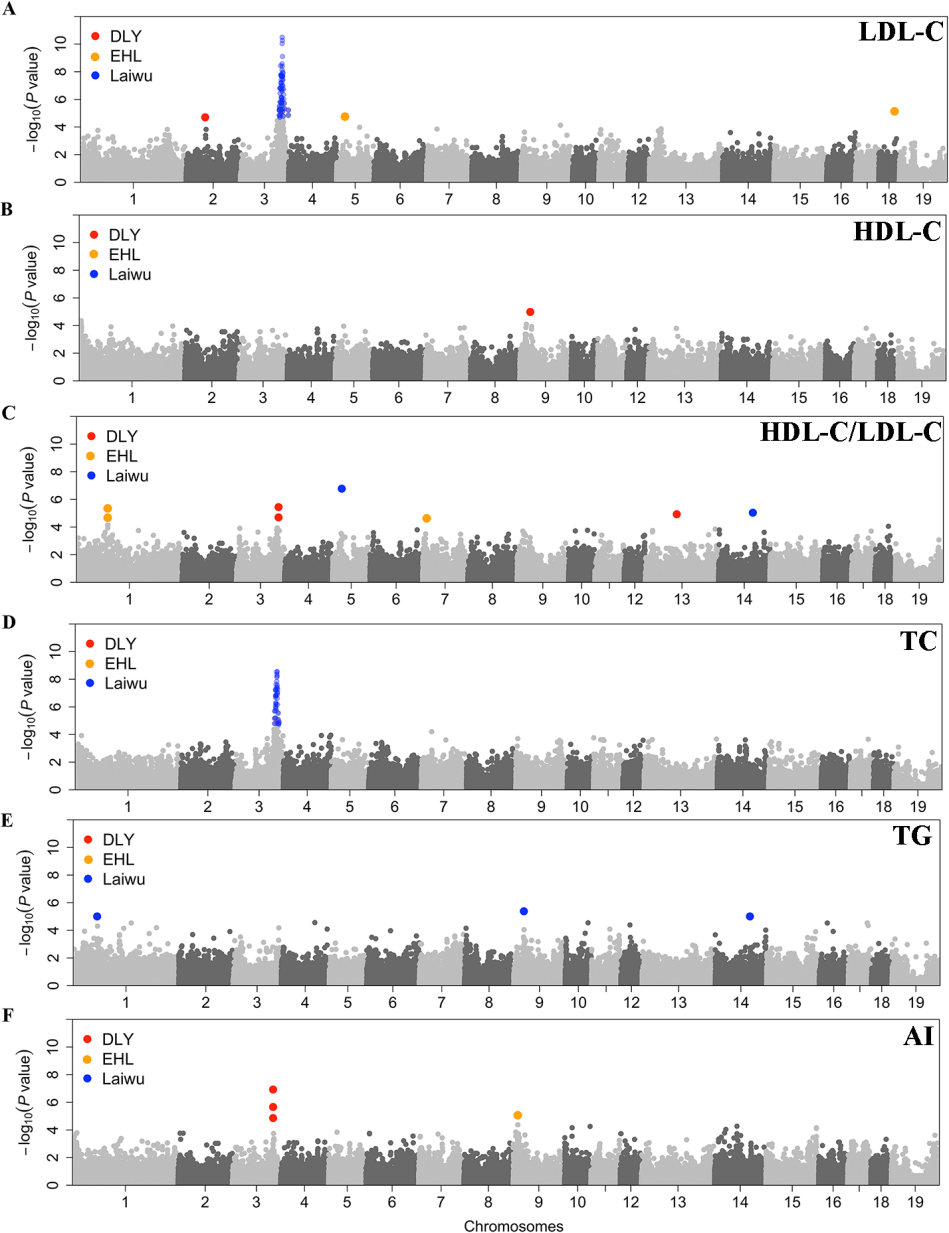
Manhattan plots of GWAS for blood lipids in the Laiwu, DLY and Erhualian populations. SNPs surpassing the threshold of suggestive significance in the three populations are denoted with different colors (red for DLY, orange for EHL and blue for Laiwu). (A-F) for LDL-C, HDL-C, HDL-C/LDL-C, TC, TG and AI, respectively.

As presented in **S4 Table**, a total of 61 SNPs located on SSC3 showed association signals for LDL-C in the Laiwu population. All these SNPs are located in a region of 116.38 to 143.79 Mb, which has been reported to harbor QTL for LDL-C in pigs [10, 22]. The most significant SNP was found at SSC3: 125,211,999 bp (*P* = 3.25 × 10^-11^, **Fig 1A**). In the DLY population, only one SNP at SSC2: 60.34 Mb achieved the suggestive significance level for LDL-C (*P* = 2.15 × 10^-5^). In the Erhualian population, we identified 4 SNPs surpassing the suggestive significance level, including 3 SNPs on SSC18 and one SNP on SSC5.

#### High density lipoproteins

We only identified one SNP at SSC9: 18.74 Mb that surpassed the suggestive significance level in the DLY population (*P* = 1.34 × 10^-5^, **Fig 1B**). In Laiwu and Erhualian pigs, no HDL-associated SNP was identified (**S4 Table**).

#### HDL-C/LDL-C

As shown in **S4 Table**, we detected 6 genomic loci that were significantly associated with HDL-C/LDL-C, including 2 loci at SSC5: 25.19 Mb and SSC14: 109.02 Mb in Laiwu pigs, 2 loci at SSC3: 123.04-123.39 Mb and SSC13: 84.66Mb in the DLY population, and 2 loci at SSC1: 94.11-95.10 Mb and SSC7: 7.47 Mb in the Erhualian population (**Fig 1C**).

#### Total cholesterol

We identified 39 TC-associated SNPs at SSC3: 118.61-131.79 Mb (*P* = 1.76 × 10^-5^ to 2.72 × 10^-9^) in Laiwu pigs (**S4 Table**). In the DLY population, one SNP MARC0085934 at SSC3: 126,039,599 bp was detected to significantly associate with TC (*P* = 1.22 × 10^-5^). However, no TC-associated SNPs were identified in the Erhualian population (**Fig 1D**).

#### Triglycerides

We found a total of 3 SNPs that were significantly associated with TG in the Laiwu population, including one SNP at SSC1: 74.02 Mb, one SNP at SSC9: 32.88 Mb and one SNP at SSC14: 107.03 Mb. No TG-associated SNPs were detected in the DLY and Erhualian populations (**Fig 1E**).

#### Atherosclerosis index

As presented in **S4 Table**, we did not detect any significant SNPs for AI in Laiwu pigs. But we identified 3 SNPs at SSC3: 123.04-123.39 Mb that were significantly associated with AI in the DLY population (*P* = 8.08 × 10^-6^ to 2.78 × 10^-7^). Further, we detected another AI-associated SNP at SSC9: 14,306,925 bp in the Erhualian population (*P* = 1.19 × 10^-5^, **Fig 1F**).

### Novel loci identified by the meta-analysis

**Fig 2:**
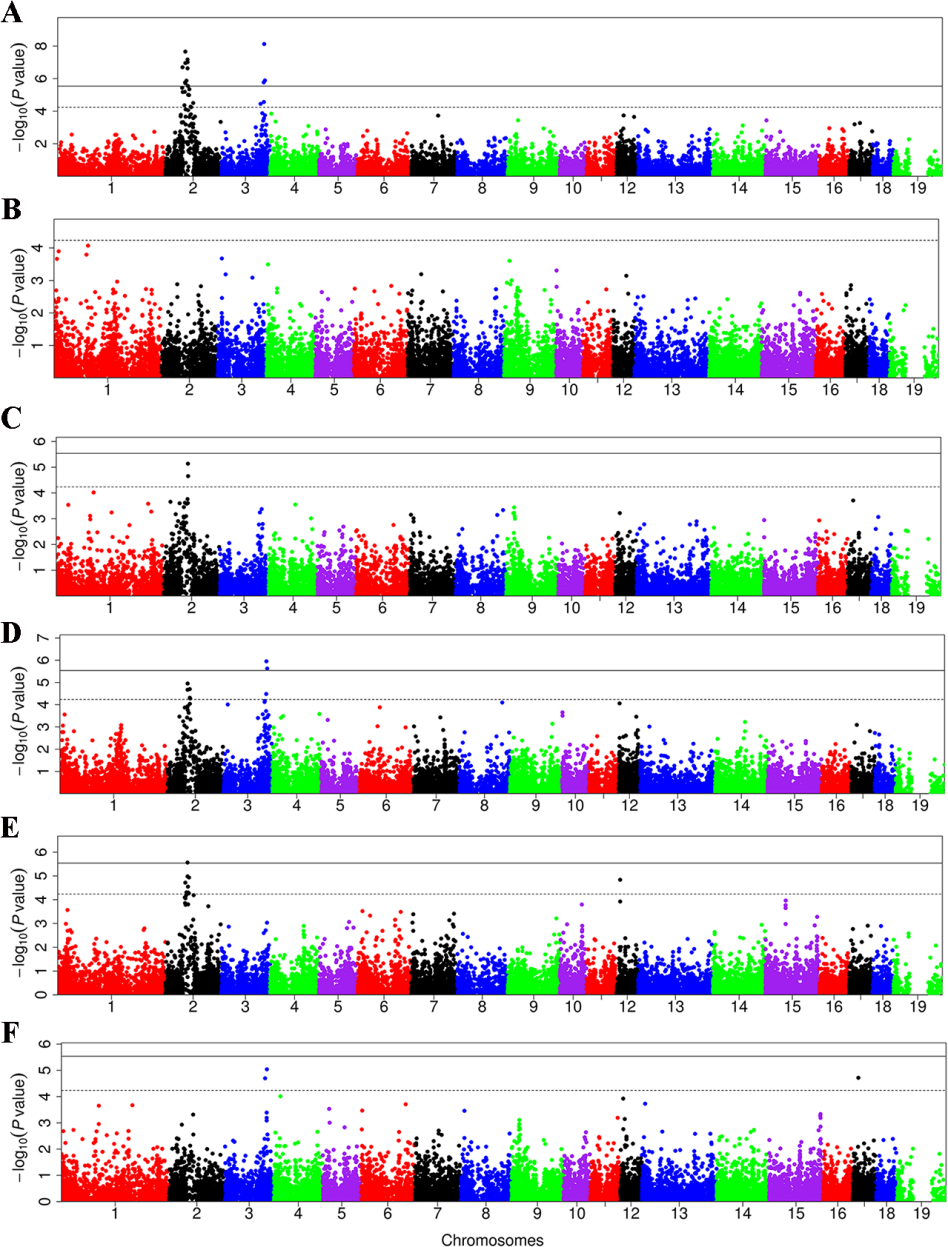
Manhattan plots of meta-analysis of GWAS for blood lipid traits in the five experimental populations. The x-axis shows chromosomal positions, and y-axis shows negative log_10_ *P* values. The solid and dashed lines indicate the thresholds of genome-wide and suggestive significance, respectively. Panels (A) to (F) represent the meta-analysis results for LDL-C, HDL-C, HDL-C/LDL-C, TC, TG and AI, respectively.

To identify novel significant loci, we performed a meta-analysis in the five pig populations. The results are shown in **S5 Table**. We detected a total of 51 SNPs within 9 genomic regions that were significantly associated with blood lipid traits (**Fig 2**). Eighteen out of the 51 SNPs surpassed the genome-wide significance level. Of the 9 genomic loci, to our knowledge, two loci (SSC2: 58.58-69.29 Mb and SSC12: 10.85 Mb) are reported to associate with TG for the first time (**Fig 2E**). SSC2: 58.58-66.37 Mb, SSC17: 16.77 Mb and SSC2: 70.54-71.04 Mb showed association with TC, AI and HDL-C/LDL-C, respectively. Moreover, the locus on SSC3 was evidenced to associate with LDL-C, TC and AI in the meta-analysis (**Fig 2**).

### LDLA mapping result for blood lipid traits with SNPs on SSC3

**Fig 3:**
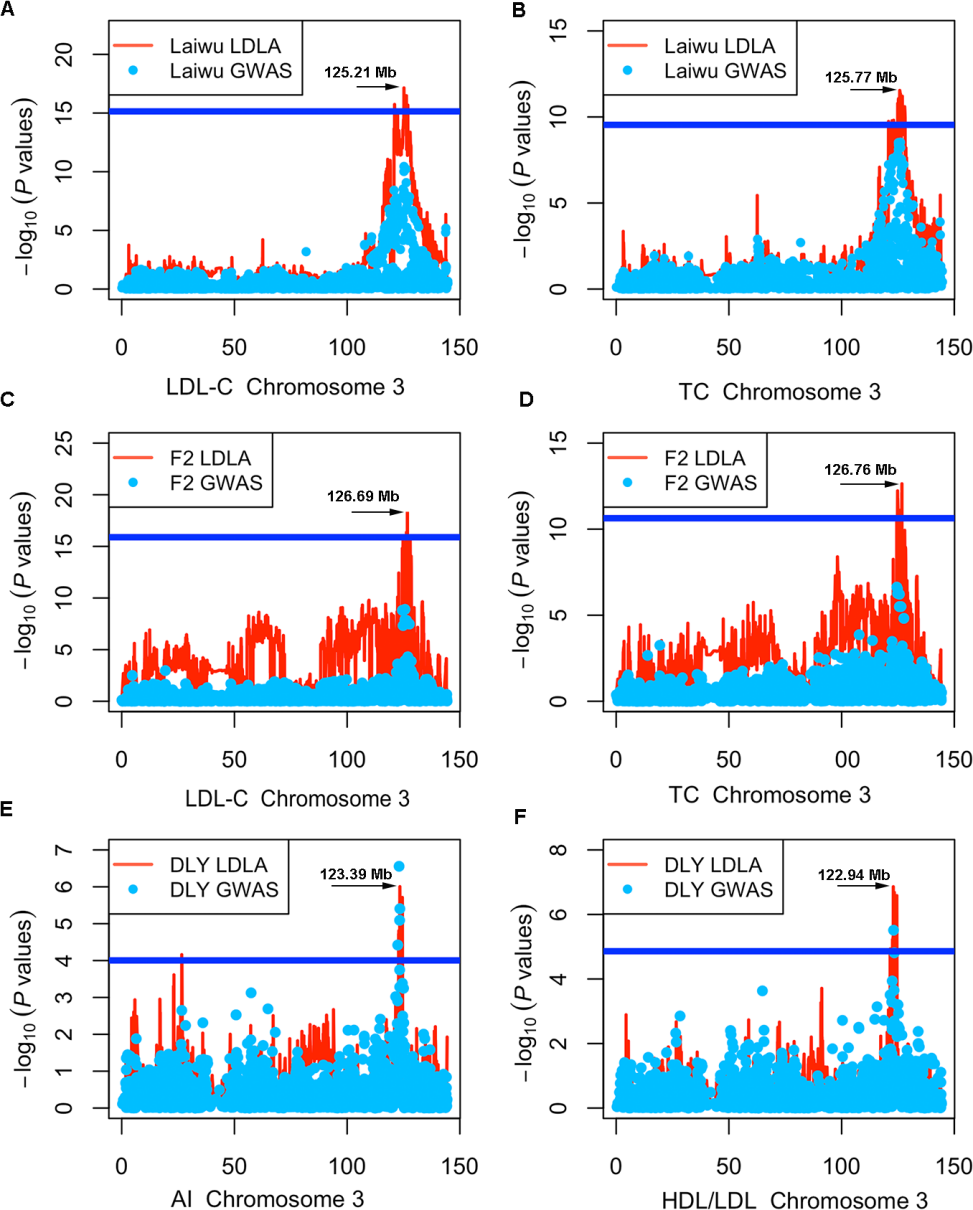
LDLA analysis for blood lipid traits with the SNPs on SSC3 in the Laiwu, F_2_ and DLY populations. The x-axis indicates the SNP positions on SSC3 (Mb), and y-axis shows negative log_10_ (*P* values) from haplotype-based association study. Blue dots show the qualified SNPs in GWAS, and red lines represent the haplotype. The horizontal line indicates the 95% of confidence interval by LOD drop off 2 from the most significant haplotype. (A-B) for LDL-C and TC in the Laiwu population; (C-D) for LDL-C and TC in the F_2_ population; (E-F) for AI and HDL-C/LDL-C in the DLY population.

We have previously tested the causality of *LDLR*, a promising candidate gene for the significant locus on SSC2, for serum lipids [11]. Here, we made a close examination on the SSC3 locus that harbored a cluster of significant SNPs for blood lipids in Laiwu, F_2_ and DLY pigs (**Fig 1**). Our haplotype-based association study (LDLA mapping) showed the most significant haplotypes around the 125.0-127.0 Mb region on this chromosome, which encompassed a highly plausible causative gene: *APOB* (**Fig 3**). The most prominent SNP (MARC0083986) and haplotype that we identified are all proximal to the *APOB* gene in Laiwu pigs.

### Associations of *APOB* polymorphisms with blood lipid traits

**Fig 4:**
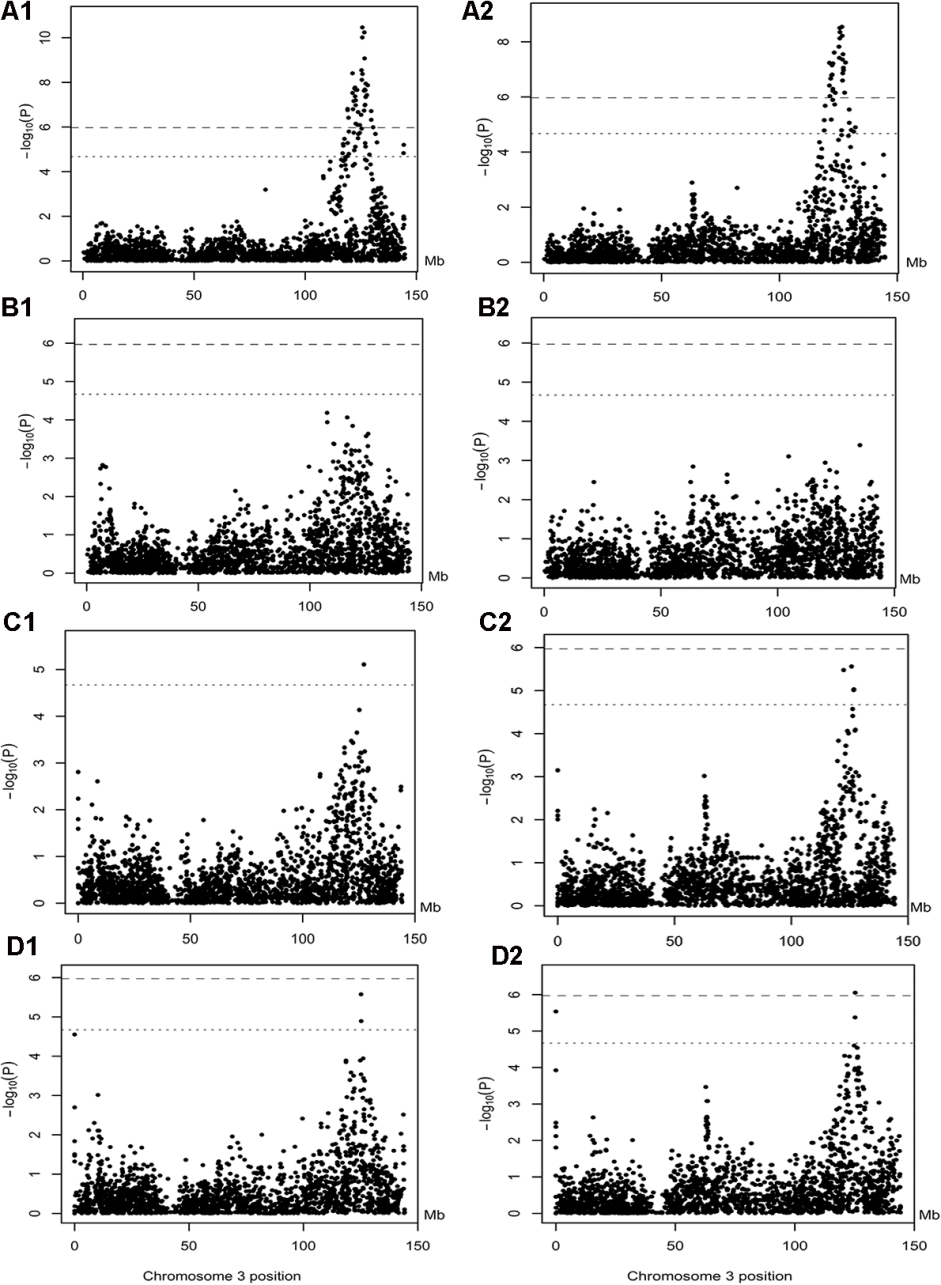
The results of F-drop test in Laiwu pigs. The x-axis shows SNP positions on SSC3 (Mb); the negative log_10_ (*P* values) is presented on the y-axis. The horizontal dotted and dashed lines indicate the thresholds of suggestive and genome-wide significance, respectively. A1 and A2 show the GWAS results for LDL-C and TC in the Laiwu population with all qualified SNPs on SSC3, in which the genotypes of 9 polymorphisms were also included; B1, C1 and D1 show the *F*-drop test results for LDL-C, in which the genotypes of indel, NW_003613573.1:g.20713A>G and NW_003613573.1:g.48834A>T were included as a fixed effect in the model, respectively; B2, C2 and D2 present the *F*-drop test results for TC.

We screened exonic variants of the *APOB* gene by Sanger sequencing of 15 F_0_ founder animals of the F_2_ population. Blasting of the flanking sequence of the nine polymorphisms against the human reference genome at http://asia.ensembl.org/index.html revealed that the indel and NW_003613573.1:g.20713A>G variants have no hits with human *APOB* gene and the other seven are within the human *APOB* gene. Furthermore, we blasted the indel and NW_003613573.1:g.20713A>G variants against the pig reference genome sequence in the Ensembl database and the Wuzhishan genome, a Chinese pig genome [23]. The blasting result showed that the two polymorphisms should be in the upstream of the pig *APOB*. As a result, we identified 8 SNPs and an indel, of which two are tested in the upstream of the *APOB* gene and the others reside in the coding region of this gene. To assess the associations of these *APOB* polymorphisms with blood lipids, we firstly performed the standard association test in the Laiwu and F_2_ populations (**Table 2**). The indel, NW_003613573.1:g.20713A>G and NW_003613573.1:g.48834A>T variants showed significant associations with LDL-C in Laiwu pigs (*P* < 2.12 × 10^-5^). The association was also observed between the indel and NW_003613573.1:g.20713A>G with TC in the Laiwu population. To further evaluate the causality of the indel, NW_003613573.1:g.20713A>G and NW_003613573.1:g.48834A>T variants for LDL-C and TC in Laiwu pigs, we performed the *F*-drop test. Only in the case of the indel mutation, the association signals for LDL-C and TC vanished completely in this population (**Fig 4**). None of these variants was associated with LDL-C and TC in the F_2_ population (*P* > 2.45 × 10^-5^).

**Table 2:** Single marker association test of the 9 polymorphisms with LDL-C and TC in the Laiwu and F_2_ populations.

Population	Variant	MAF	HWE^a^	Single marker association test	
				LDL-C (*P* value)	TC (*P* value)
**Laiwu (n = 300)**	NW_003613573.1:g.19863_20286delinsCATCGTACCGA	0.35	6.00 × 10^-3^	8.15 × 10^-8^	1.34 × 10^-7^
	NW_003613573.1:g.20713A>G	0.44	3.00 × 10^-3^	8.05 × 10^-7^	2.05 × 10^-5^
	NW_003613573.1:g.48236G>C	0.26	0.63	0.43	0.06
	NW_003613573.1:g.48834A>T	0.49	0.1	1.87 × 10^-7^	9.00 × 10^-4^
	NW_003613573.1:g.48943G>C	0.17	1	0.19	4.00 × 10^-3^
	NW_003613573.1:g.50536T>C	0.05	0.98	0.39	0.58
	NW_003613573.1:g.53468G>A	0.26	0.57	0.42	0.06
	NW_003613573.1:g.53546G>A	0.06	0.7	0.81	0.31
	NW_003613573.1:g.58318C>G	0.15	0.64	0.75	0.14
**F2 (n = 729)**	NW_003613573.1:g.19863_20286delinsCATCGTACCGA	0.27	0.03	0.18	0.22
	NW_003613573.1:g.20713A>G	0.29	0.77	1.00 × 10^-3^	7.00 × 10^-4^
	NW_003613573.1:g.48236G>C	0.13	7.00 × 10^-3^	0.01	7.00 × 10^-3^
	NW_003613573.1:g.48834A>T	0.5	6.00 × 10^-4^	0.07	0.02
	NW_003613573.1:g.48943G>C	0.34	2.00 × 10^-3^	0.25	0.31
	NW_003613573.1:g.50536T>C	0.26	3.00 × 10^-3^	0.32	0.38
	NW_003613573.1:g.53468G>A	0.19	0.18	0.21	0.03
	NW_003613573.1:g.53546G>A	0.09	0.17	0.57	0.91
	NW_003613573.1:g.58318C>G	0.44	2.00 × 10^-4^	0.01	9.00 × 10^-3^

^a^HWE, Hardy Weinberg Equilibrium.

## Discussion

In this study, we investigated the genetic basis of blood lipid traits in more than 2,400 pigs from five populations through GWAS and meta-analysis. To our knowledge, our sample size is the largest one in the GWAS for porcine blood lipid traits. We identified a total of 22 genomic regions that were significantly associated with blood lipid levels in the five populations. The tested populations shared few genomic loci. This provides strong evidence of a substantial level of genetic heterogeneity for pig serum lipids. Of the 22 genomic regions, only 6 were identified in more than one population, and 16 loci were detected in a single population.

### The genomic regions associated with blood lipid traits shared by multiple pig populations

Several significantly associated genomic regions identified in this study replicated the findings in the previous reports. SSC3: 124.0-126.0 Mb was associated with LDL-C and TC in Laiwu pigs in this study. This region was also associated with LDL-C and TC in the F_2_ population [10]. Moreover, Manunza et al. [24] and Gallardo et al. [22] detected significant associations for LDL-C and TC in Duroc pigs in this region. SSC2: 60.34 Mb was associated with LDL-C in the DLY population (*P* = 2.15 × 10^-5^). This region has also been shown to be significantly associated with LDL-C in Sutai and F_2_ pigs [10]. SNP MARC0010324 (SSC9: 18.74 Mb) had significant association with HDL-C in the DLY population. Interestingly, Gallardo et al. [22] also detected a QTL for HDL-C around this region in Duroc pigs. The genomic regions associated with TG on SSC12 and SSC14 were consistent with the findings by Gallardo et al. [22] and Manunza et al. [24], respectively. Further fine-mapping would be needed to examine whether allelic heterogeneity, a frequent feature of polygenic traits in humans [25–27], exists or not in the above-mentioned genomic loci shared by multiple populations.

### Population-specific genomic regions associated with porcine blood lipid traits

Of 22 significant loci that we identified, 16 were detected in a single population, suggesting the existence of population heterogeneity (**Fig 1**). For certain, we cannot rule out the possibility that different sample sizes and phenotypes measured at different ages may cause different GWAS results in each population. At the SSC2 locus, we have observed the association signals for LDL-C and TC in both F_2_ and Sutai populations. We highlight *LDLR* as a strong candidate gene at this locus, and further show that different causative variants in this gene likely underlie phenotypic variation in the two populations [11]. Manunza et al. have reported age-specific genetic determinants for porcine serum lipid traits [24]. In humans, most of significant loci for blood lipids were different among different aged individuals or ethnic groups [28, 29]. Therefore, further investigations are warranted to test if population-specific causative variants underlie the significant loci identified in this study.

### Possible pleiotropic QTL

We found two genomic regions that were significantly associated with more than one trait in GWAS and meta-analysis. SSC2: 60.0-71.0 Mb was associated with LDL-C, TC, TG and HDL-C/LDL-C in the meta-analysis (**S5 Table**), and SSC3: 121.0-127.0 Mb was related to LDL-C, TC and AI. The finding can be explained by: 1) QTL with a common variant with pleiotropic effects; 2) Strong correlation between the associated phenotypes (**S2 Fig**), as previously observed between TC and LDL-C, and between TC and HDL-C [9]; 3) The two regions contain more than one QTL influencing blood lipid traits.

### Plausible candidate genes at the identified loci

To identify candidate genes at the genomic loci surpassing the genome-wide significance level, we searched annotated genes with functional relevance to blood lipids or lipid metabolism in an interval of 5 Mb centered at the top SNP. At the SSC3 locus, *NCOA1*, *KLHL29*, *APOB*, *C2ORF43* and *TTC32* are promising candidate genes. *NCOA1* encodes a transcriptional coactivator for steroid. It controls energy balance between white and brown adipose tissues [30]. Both *KLHL29* and *APOB* have been reported as candidate genes for blood lipids [31]. The function of *KLHL29* is not yet known, but this gene has been implicated in coronary heart disease [31]. *APOB* encodes the main apolipoprotein of chylomicrons and low density lipoproteins. It appears to be a strong candidate gene affecting LDL-C and TC in both humans and pigs [9, 22, 32–34]. Several polymorphisms at the *APOB* locus, including the 3611 MspI polymorphism in the promoter region and a truncating mutation, have been shown to associate with dyslipidemia in humans [32, 35]. *C2ORF43* is functionally related to defective apolipoprotein b-100 and coronary heart disease [36]. Lu et al. reported that *TTC32* is a strong candidate gene for coronary artery disease by GWAS [37]. *SMARCA4*, *LDLR* and *INSR* play a role in lipid metabolism [26, 38]. These genes reside in the genomic region of SSC2: 64.97-70.25 Mb that was significantly associated with LDL-C and TC in this study. We have investigated that *LDLR* is a causative gene for LDL-C and TC in the Sutai population, but allelic heterogeneity exists between different populations [11]. *TAC3* and *GPR182* have been investigated as candidate genes for the locus on SSC5 [39, 40], where MARC0010341 at 25.19 Mb (*P* = 4.75 × 10^-7^) was significantly associated with HDL-C/LDL-C in this study.

### Associations of *APOB* polymorphisms with porcine blood lipid levels

We identified 9 polymorphisms nearby or within the *APOB* gene in the founders of F_2_ intercross. Standard association test and *F*-drop test strongly suggested that the indel is a strong candidate causative mutation for LDL-C and TC at the SSC3 locus in Laiwu pigs. This conclusion was strengthened by the observation that the indel was not found or was segregated at very low frequency in the Sutai, Erhualian and DLY populations (**S6 Table**), in which the effect of the SSC3 locus was not identified. Unexpectedly, no significant association was observed between the indel and LDL-C or TC in the F_2_ population in which this variant was segregating at a MAF of 0.27 (**Table 2**). This observation could be explained by 1) population heterogeneity exists between the two populations. A line of supporting evidence comes from a finding that the most significant haplotype for LDL-C and TC was not identified in the same region on SSC3 in the Laiwu (125.21 Mb and 125.77 Mb) and F_2_ (126.69 Mb and 126.76 Mb) populations. So variants in different genes may cause the association signal on this chromosome; 2) the indel is not the causative mutation for LDL-C and TC. It is just in high linkage-disequilibrium with the real causative mutation.

## Conclusions

In conclusion, we performed GWAS to identify genomic loci associated with blood lipids in five diverse pig populations. Our results highlight a substantial level of population heterogeneity for genetic components of porcine blood lipid traits. Our previous and current studies collectively suggest that population-specific variants may cause the QTL effect on SSC2 and SSC3 identified in multiple pig breeds. *APOB* is a promising candidate gene for LDL-C and TC at the SSC3 locus. Further fine mapping and functional assays are required to confirm the causality of the indel variant in the *APOB* gene for LDL-C and TC.

## Supporting Information

S1 Fig The genotyping results of the indel by agarose gel electrophoresis. QQ, Qq and qq stand for homozygote of normal, heterozygote and homozygote of indel, respectively.
(TIF)

S2 Fig Magnitudes of correlations between the six blood lipid traits. The dots indicate the significant correlation coefficients between each pair of traits. Their sizes and colors represent the degree and direction (positive and negative) of the correlations, respectively.
(TIF)

S3 Fig Quantile-quantile (Q-Q) plots of the observed *P*-values versus the expected *P*-values of association in GWAS for blood lipids. (A1-F1) For LDL-C, HDL-C, HDL-C/LDL-C, TC, TG and AI in the Laiwu population; (A2-F2) For LDL-C, HDL-C, HDL-C/LDL-C, TC, TG and AI in DLY pigs; (A3-F3) For LDL-C, HDL-C, HDL-C/LDL-C, TC, TG and AI in the Erhualian population.
(TIF)

S1 Table PCR primers for identification of polymorphisms of the *APOB* gene. (XLSX)

S2 Table Primer and probe sequences for genotyping the 9 *APOB* variants. (XLSX)

S3 Table Summary of phenotypic values for blood lipid traits in the Laiwu, DLY and Erhualian populations. (XLSX)

S4 Table The SNPs above the suggestive significance level for blood lipid traits identified in the Laiwu, Erhualian and DLY populations by GWAS. (XLS)

S5 Table The SNPs that achieved the suggestive significance level and were identified in the five populations by meta-analysis of GWAS. (XLS)

S6 Table Phenotypic least square means (LSM) for genotypes of the nine *APOB* polymorphisms in the five populations. (XLSX)
