# Supplementary figures and images for: Genome-Wide Association Analysis for Blood Lipid Traits Measured in Three Pig Populations Reveals a Substantial Level of Genetic Heterogeneity
Source: PLoS One. 2015 Jun 29;10(6):e0131667. doi: 10.1371/journal.pone.0131667 (PMC4488070; doi:10.1371/journal.pone.0131667)

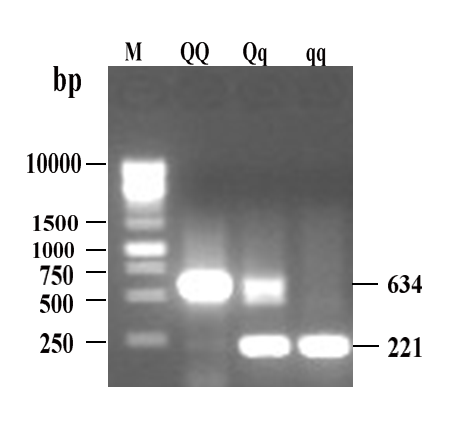

Supplement: S1 Fig — QQ, Qq and qq stand for homozygote of normal, heterozygote and homozygote of indel, respectively. (TIF) [file pone.0131667.s001.tif]

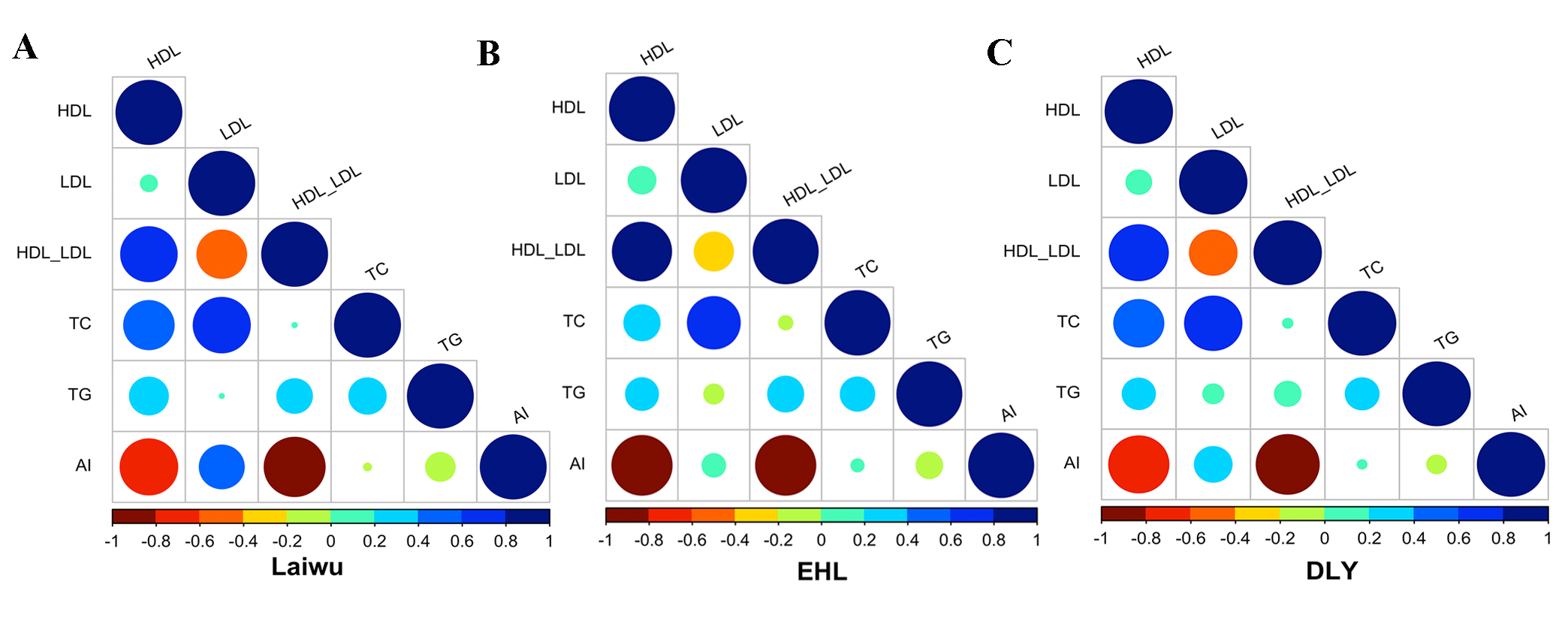

Supplement: S2 Fig — The dots indicate the significant correlation coefficients between each pair of traits. Their sizes and colors represent the degree and direction (positive and negative) of the correlations, respectively. (TIF) [file pone.0131667.s002.tif]

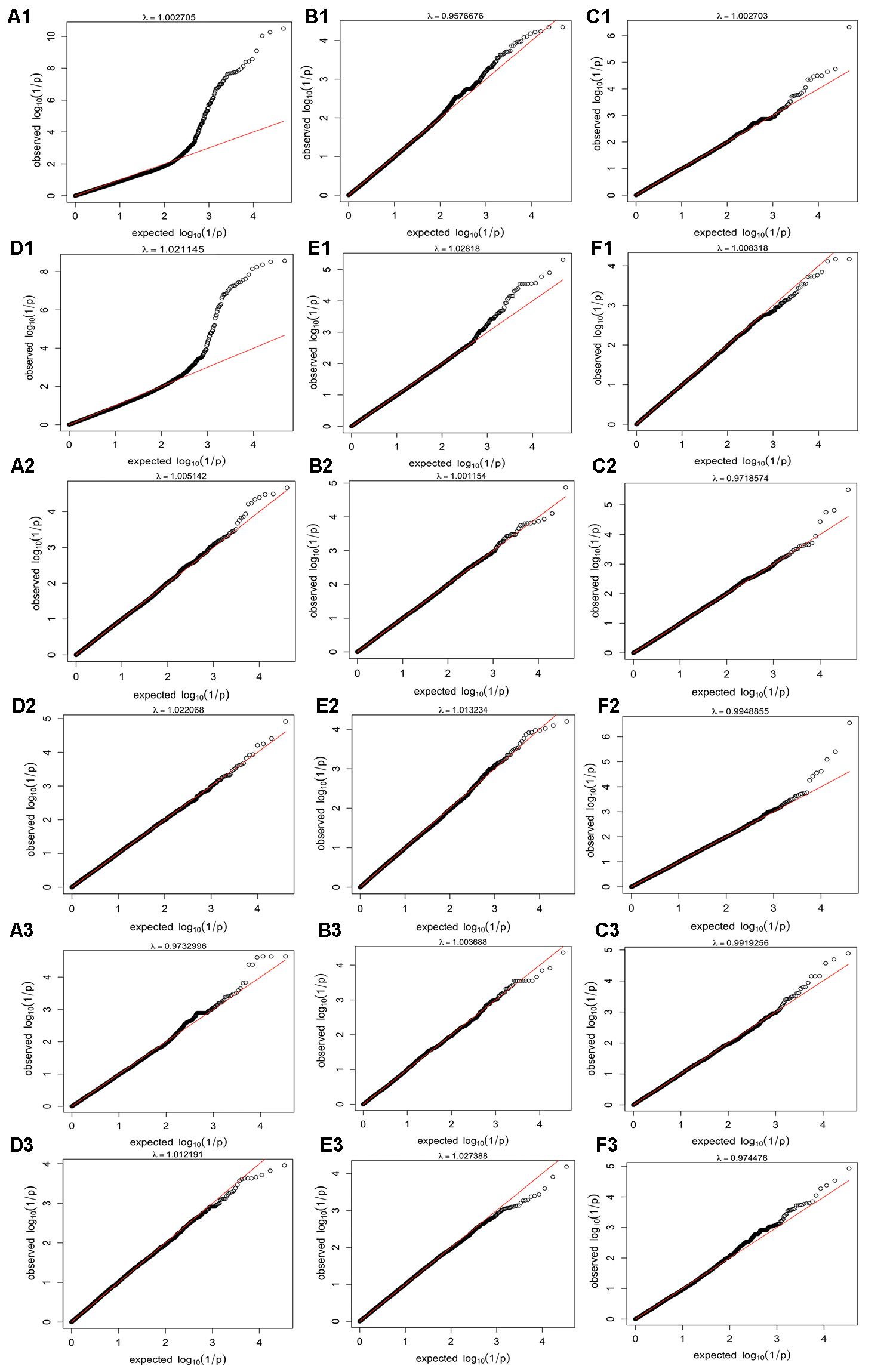

Supplement: S3 Fig — (A1-F1) For LDL-C, HDL-C, HDL-C/LDL-C, TC, TG and AI in the Laiwu population; (A2-F2) For LDL-C, HDL-C, HDL-C/LDL-C, TC, TG and AI in DLY pigs; (A3-F3) For LDL-C, HDL-C, HDL-C/LDL-C, TC, TG and AI in the Erhualian population. (TIF) [file pone.0131667.s003.tif]
